# Evaluation of neuropathy during intensive vincristine chemotherapy for non-Hodgkin's lymphoma and Acute Lymphoblastic Leukemia

**Published:** 2013-10-22

**Authors:** M Dorchin, R Masoumi Dehshiri, S Soleiman, M Manashi

**Affiliations:** 1 Radiation Oncologist, Oncology Department, Dezfol University of Medical Sciences. Dezfol. Iran.; 2 HematoOncologist, Oncology department, Al-Bairouni University Hospital, Medical faculty, Damascus University, Damascus, Syria.; 3 Health Policy Research Center, Shahid Sadoughi University of Medical Sciences and Health Services, Yazd, Iran.

**Keywords:** Vincristine, Neuropathy, Acute Lymphoblastic Leukemia, non-Hodgkin's lymphoma

## Abstract

**Back ground:**

Vincristine (VCR), is a chemotherapy drug, useful in the treatment of leukemia, lymphoma and solid tumor and it is a potent neurotoxin and sensory neuropathy drug which a common behavioral toxicity of this drug. Neuropathy is common squeal of intensive chemotherapy protocols that contain vincristine and corticosteroids.

**Materials and Methods:**

This study was a retrospective and descriptive study of neuropathy during in chemotherapy program with vincristine for patients with non-Hodgkin's lymphoma (NHL) and Acute Lymphoblastic Leukemia (ALL). Data was analyzed by spss Version16 software.

**Results:**

From total of 51 cases, 23 patients had vincristine neuropathy (45%). Patients with visceral neuropathy have shown ileus, constipation in 13 patients (25%), occasionally severe diarrhea 11 (21%), mild diarrhea 7 (13.7%) and transient diarrhea in 16 patients (31%).

Motor neuropathy were found in one patient with Bell, s palsy (1.9%) and one patient with Hoarseness. 12 patients (23.5%) had some type of complication together with sensory peripheral neuropathy.

**Conclusion:**

Almost half of patients with vincristin chemotherapy had neuropathy and the mean age of patients with neuropathy was 12.3 years.

## Introduction

Vincristine(VCR) is a member of vinca alkaloid family and is indicated for the treatment of Hodgkin, non Hodgkin's lymphoma and particularly in pediatric cancers either by itself or in combination with other antitumor agents(1,2). 

chemotherapy-induced peripheral neuropathy (CIPN) is a dose-limiting toxicity of Chemotherapy that often develops in response to administration of various drugs, including, molecularly targeted therapeutic agents bortezomib, taxanes (paclitaxel, docetaxel), platinum compounds, platinum-containing drugs (cisplatin, carboplatin, oxaliplatin), vinca alkaloids (vincristine), thalidomide, lenalidomide, and epothilones (3). The cytotoxicity induced by VCR is based on well established pharmacologic properties that include, binding to tubulins and disrupting microtubules formation in mitotic spindles and thus preventing cell division (4, 5).

Vincristine is a naturally occurring vinca alkaloid used in various chemotherapy regimens. Neurotoxicity is a known and commonly encountered side effect of vincristine. Peripheral neuropathy is the most common form of vincristine neuropathy, whereas central effects are rarer (6).

In fact, CIPN may represent the initial stage in development of neuropathic pain. Although the symptoms of CIPN are diverse, the condition consist ently reduces patient quality of life (QOL). 

So, effective strategies for preventing or treating CIPN remain elusive. To identify significant predictors for CIPN which would contribute for improving the QOL (quality of life) among chemotherapy patients (7). Here we designed a comprehensive study to evaluated neuropathy induced by VCR in patients with NHL and ALL.

## Materials and Methods

The study was retrospective and descriptive study of neuropathy during chemotherapy program with vincristine in patients with NHL and ALL. We performed evaluation of neuropathy during a chemotherapy program with vincyristin for patients with intermediate and high-grade NHL and ALL that was carried in Al-Bairouni hospital in Damascus, Syria from June 2007 to June 2009. The study population consisted of 51 patients, 30 patients with NHL and 21 with ALL.

Inclusion criteria:

The patients' clinical staging was determined according to the text classification. Diagnostic work-up and staging procedures at the presentation time included patient medical history and complete physical examination, Complete Blood Count (CBC), serum biochemistry, erythrocyte sedimentation rate (ESR), bone marrow aspiration and biopsy (BMA&BMB), chest X-ray, computed tomography (CT) scan of the chest, CT or ultra sonography of the abdomen and the pelvic, and other procedures if it was nesseory.

Exclusion criteria were diabetes, known history of neuropathy before ALL and NHL diagnosis, radiation treatment, bone marrow transplantation and children with trisomy 21.

All pathology slides were reviewed by pathologists of the center Al-Bairouni hospital. Moreover, immune histochemical studies such as leucocytes common antigen (LCA), cluster of differentiation (CD), CD15, CD20, and other markers, were performed to determine the subtype of non-Hodgkin's lymphoma (NHL). Classification by the Revised European-American Lymphoma (REAL)/the World Health Organization (WHO) was used for histopathology sub classifiation.

Vincristine was administered by bolus injection in NHL at dose of 1.4mg/m^2^ and for ALL with 1.5mg/m2 and total dose of 2.0 mg/m2 per week; the maximum dose of vincristine was not arbitrarily limited. Patients administrated intravenous every week.

The dose for weekly infusion was determined based on pharmacokinetic parameter. The diagnosis of neuropathy is established by abnormalities in clinical history, examination, and electromyography (EMG) for muscle denervation. Patients with sensory peripheral neuropathy were classified according to grades: 

Grade 0: Normal

Grade 1: Asymptomatic; loss of deep tendon reflexes or paresthesia (including tingling) but not interfering with function.

Grade2: Sensory alteration or paresthesia (including tingling), interfering with function but not interfering with Activities of Daily Living (ADL).

Grade3: Sensory alteration or paresthesia interfering with ADL.

Grade 4: Disabling

Grade 5: Death

**Statistical Analysis**

Data was analyzed by spss Version16 based on aims using descriptive statistics. 

## Results

**Figure 1:**
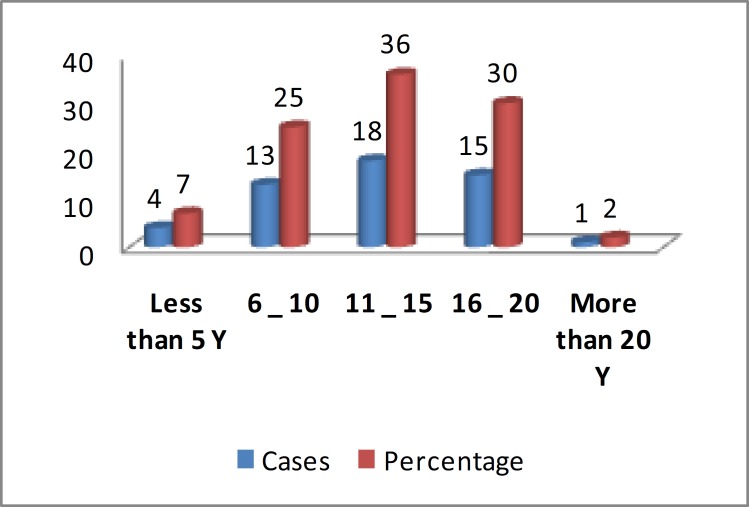
Incidence according to the Age group. (y=years)

During the evaluation of neuropathy in chemotherapy program with vincyristin, 51 patients (31 males (61%)) were assessed. The ratio of male to female was 3:2. 31(59%) of patients were NHL and 21(41%) of patients were ALL. The mean age of patients was 12.3 (range: 5 - 20 years) (Figure1). 

Anemia (67%), fever (61%) and weakness (52%) were the most common presenting symptoms in patients with NHL and ALL. 

Lactate Dehydrogenase (LDH), as an important prognostic factor in non-Hodgkin's lymphoma was elevated in 9 patients (32.1%). Anemia was seen in 20 patients (66.6%). In the majority of patients, other laboratory tests such as platelets, white blood cell count and liver enzymes were normal. From total patients 51cases, 23 patients were vincristine neuropathy (45%), and 11 cases were mild (21%). patients with visceral neuropathy have shown ileus, constipation in 13 patients (25%), occasionally severe diarrhea in 11 (21%), mild diarrhea in 7 (13.7%) and transient diarrhea in 16 patients (31%). Motor neuropathy was found in one patient with Bells palsy (1.9%) and one Hoarseness who had only jaw pain (1.9%). 12 patients (23.5%) had some type of complication together. According to sensory neuropathy grading, 33 patients (64%) were Grade 0, 9 patients (17.6%), 7 (13.7%), 2 (3.9%) were grade 1, 2, 3 and none of patients were grade 4 and 5 (Table II).

The mean age of patients with motor neuropathy and visceral neuropathy was 14.5 and 16 years, respectively. In case of grade 2, the dose is one-half in only 7 cases which had developed complications, causing amputation that induced by vincristine. In mild visceral neuropathy the dose is half that in two cases the condition is cured and the treatment is in progress. Visceral and severe neuropathy in all cases where the drug has been completely cut off marks, have been removed. Motor neuropathy, the drug was 

discontinued completely in all cases, despite the gradual improvement of symptoms, complete remission has been achieved.

**Table I:** Grading and Results of sensory peripheral neuropathy assessments during Vincristine therapy

**Grade**						
**Adverse event**	**0**	**1**	**2**	**3**	**4**	**5**
**Neuropathy-sensory**	Normal	Asymptomatic; loss of deep tendon reflexes or paresthesia (including tingling) but not interfering with function	Sensory alteration or paresthesia (including tingling), interfering with function but not interfering with ADL	Sensory alteration or paresthesia interfering with ADL	Disabling	Death
**In this study**	33(64%)	9(17.6%)	7(13.7%)	2(3.9%)	0	0

ADL, activities of daily living

**Table II:** Clinical complications for vincristine chemotherapy for non-Hodgkin's lymphoma and Acute Lymphoblastic Leukemia

	**Case**	**Percentage(%)**
**Neuropathy(total)**	23	45
**Sensory Neuropathy(sever)**	5	9.8
**Hyperesthesia**	7	13.7
**Sensory Neuropathy(mild)**	11	21.5
**Visceral Neuropathy(sever) **	11	21.5
**Visceral Neuropathy(mild) **	7	13.7
**Motor neuropathy**	16	31
**Bell, s palsy**	1	1.9
**Hoarseness**	1	1.9
**some type of complication together**	12	23.5

## Discussion

Vincristine administration as part of a chemotherapy regimen is known to cause primary toxic myopathy, myelopathy, and peripheral neuropathy (8, 9). DeAngelis et al reported that intensive administration of intravenous vincristine (total dose of 2 mg/m2 per week), and corticosteroids for 12 weeks to patients with non-Hodgkin’s lymphoma resulted in neuropathy and myelopathy in all 27 evaluable studied patients. Symptoms were most apparent in the distal extremities, involving mainly the extensor muscles (10). Correale et al reported toxic polyneuropathy in 39 (3.9%) of 989 patients with lymphoma undergoing cytotoxic treatment (11).

Peripheral neuropathy can be due to axonal loss, demyelinating injury, or both. Vincristine-induced neuropathy usually presents with axonal loss on EMG, leading to loss of amplitude of nerve action potentials and evidence of denervation on needle examination of affected muscles (12). 

Hamilton et al demonstrated vincristine-induced peripheral neuropathy in a dog, with an EMG result consistent with muscle denervation. In most reported studies, neuropathy associated with vincristine therapy affected more than one nerve (that is, resulted in polyneuropathy), and involved the distal extremities. This patient had neuropathy involving the left suprascapular nerve only (13).

Acute leukemia is the most common malignant cancer in children and about30percent of themare included.Of these, approximately75% cases of acute leukemia accounted for, All gives lymphoblastic (14). NHL (all subtypes combined) is the sixth most common cancer (2010), accounting for 4% of all new cases. NHL is the fifth most common cancer among men (2010), accounting for 4% of all new cases of cancer in males. Among women, NHL is the seventh most common (2010), accounting for 4% of all new cases of cancer in females (15,16). 

Verstappen et al. reported that while neuropathic changes were observed in both dose intensity groups, the higher dose intensity group reported significantly more symptoms during therapy, whereas neurologic signs were significantly more prominent after a cumulative dose of 12 mg vincristine. Furthermore, off-therapy exacerbation of symptoms (24%) and signs (30%) occurred unexpectedly in that trial (17). Weintraub et al. reported that colony-stimulating factors could precipitate a severe atypical neuropathy when given in conjunction with vincristine. The development of this severe atypical neuropathy was most strongly associated with the cumulative dose of vincristine (18).

## Conclusion

Vincristine neuropathy is a common complication,which its incidence increases with age. Visceral neuropathy and motor neuropathy mainly seen in people over 50 years and people less than 20 years of age. In this study, the mean age of patients with neuropathy was 12.3 years and the mean age of patients without this complication,was 9 years. 

## Conflict of interest

The authors have no conflict of interest.

## References

[1] Fisher RI, Gaynor ER, Dahlberg S, Oken MM, GroganTM, Mize EM, Glick JH, Coltman CA Jr, Miller TP (1993). Comparison of a standard regimen (CHOP) with three intensive chemotherapy regimens for advanced non-Hodgkin's lymphoma. N Engl J Med.

[2] Kantarjian HM, Walters RS, Keating MJ, Smith TL, O'Brien S, Estey EH, Huh YO, Spinolo J, Dicke K, Barlogie B (1990). Results of the vincristine, doxorubicin, and dexamethasone regimen in adults with standard- and highrisk, acute lymphocytic leukemia. J Clin Oncol.

[3] Porter CC, Carver AE, Albano EA (2009). Vincristine induced peripheral neuropathy potentiated by voriconazole in a patient with previously undiagnosed CMT1X. Pediatr Blood Cancer.

[4] Owellen RJ, Owens AH Jr, Donigian DW (1972). The binding of vincristine, vinblastine and colchicine to tubulin. Biochem,Biophys Res Commun.

[5] Rosenthal S, Kaufman S (1974). Vincristine neurotoxicity. Ann Intern Med.

[6] 6- F, Baniasadi S, Seifi S, Fahimi F (2012). Vincristine-induced seizure potentiated by itraconazole following RCHOP chemotherapy for diffuse large B-cell lymphoma. Foroughinia, Curr Drug Saf.

[7] Y, Hosokawa T, Okamoto K, Konishi H, Otsuji E, Yoshikawa T, Takagi T (2010). Statistical identification of predictors for peripheral neuropathy associated with administration of bortezomib, taxanes, oxaliplatin or vincristine using ordered logistic regression analysis. Anti cancer Drugs.

[8] Griggs RC, Bradley WG, Shahani B, Wilson JD, Braunwald E, Isselbacher KJ Approach to the patient with neuromuscular disease. Harrison’s principles of internal medicine.

[9] Macdonald DR (2001). Neurologic complications of chemotherapy. Neurol Clin 1991;9:955-67. Ludig T, Walter F, Chapuis D, Mole D, Roland J, Blum A. MR imaging evaluation of suprascapular nerve entrapment. Eur Radiol.

[10] DeAngelis LM, Gnecco C, Taylor L, Warrell RP Jr (1991). Evolution of neuropathy and myopathy during intensive vincristine/corticosteroid,chemotherapy for non-Hodgkin’s lymphoma. Cancer.

[11] Correale J, Monteverde DA, Bueri JA, Reich EG (1991). Peripheral nervous system and spinal cord involvement in lymphoma. Acta Neurol Scand.

[12] Poncelet AN (1998). An algorithm for the evaluation of peripheral neuropathy. Am Fam Physician.

[13] Hamilton TA, Cook JR Jr, Braund KG, Morrison WB, Mehta JR (1991). Vincristine-induced peripheral neuropathy in a dog. J Am Vet Med Assoc.

[14] AG, Argyriou AA, Scopa CD, Kottorou A (2010). a new insight into the pathogenesis of chronic oxaliplatin-induced peripheral neuropathy?. Eur J Neurol.

[15] Data were provided by the Office for National Statistics on request.

[16] Data were provided by the Welsh Cancer Intelligence and Surveillance Unit on request.

[17] Cavaletti G, Frigeni B, Lanzani F, Piatti M, Rota S, Briani C et al (2007). The Total Neuropathy Score as an assessment tool for grading the course of chemotherapy-induced peripheral neurotoxicity: comparison with the NationalCancer Institute-Common Toxicity Scale. J Peripher Nerv Syst..

[18] Weintraub M, Adde MA, Venzon DJ, Shad AT, Horak ID, Neely JE, Pediatric Branch, National Cancer Institute, Bethesda, MD (1996). Severe atypical neuropathy associated with administration of hematopoietic colony-stimulating factors and vincristine. J Clin Oncol.

